# Supplementary material for: Cancer as a Chronic Illness in Colombia: A Normative Consensus Approach to Improving Healthcare Services for those Living with and beyond Cancer and Its Treatment
Source: Healthcare (Basel). 2021 Nov 29;9(12):1655. doi: 10.3390/healthcare9121655 (PMC8701263; doi:10.3390/healthcare9121655)
Supplement: Supplementary file 1 [file healthcare-09-01655-s001.zip › healthcare-1451820-supplementary.pdf]

healthcare

MDPI

Supplementary Material

Table S1. Focus group questions English and Spanish.

Focus group questions	
**English**	**Spanish**
Participants' introduction	Presentación de los participantes
We would like to hear about your experience with the illness and the treatment	Nos gustaría escuchar un poco de su experiencia con la enfermedad y el tratamiento
Did you have to experience any side effects? What was it? What did you do? Where did you go?	¿Presentaron algún efecto secundario? ¿Cuáles fueron? ¿Qué hicieron? ¿Dónde acudieron?
Did you receive information on the possible side effects?	¿Recibieron información sobre los posibles efectos secundarios?
What did you consider are the principal side effects? Which were more difficult to treat?	¿Cuáles considera usted fueron los principales efectos secundarios? ¿Cuáles encontraron más difíciles de tratar?
Did you have any difficulties due to your health system?	¿Tuvieron alguna dificultad por su sistema de salud?
Do you consider existing and unmet needs that are not covered or do not have enough attention?	¿Existe alguna necesidad que considere no está cubierta o no se le ha prestado la atención suficiente?
What strategies do you suggest for obtaining information on treatment side effects?	¿Qué estrategias sugiere para obtener información sobre los efectos secundarios?

Table S2. Focus group quotes English and Spanish.

Priorities	Focus group participant quotes	
**Mapping information resources and available support services for managing long-term cancer care**	P2: I found an advertisement (...) that gave talks for people who had (...) radiotherapies or chemotherapies, but it turns out that these talks are for a certain number of people and they don't tell everyone	P2: Encontré un volantico (...) que dictaban una charlas, para las personas que tuvieran (…) radioterapias o quimioterapias, pero resulta que esas charlas, son para determinado número de personas y no se lo dicen a todo el mundo
	P4: It is a very short-term effect. They do not tell you what will happen in the long term with your treatment as the system is so fragmented	P4: Es un efecto muy cortoplacista, no te dicen que va a pasar en el largo plazo con tu tratamiento como el sistema esta tan fragmentado
	P4: The oncologist was highly renowned, but he was a person that you couldn't ask anything about.	P4: El oncólogo era super renombrado, pero era un persona que tu no le podías preguntar nada.
	P4: There is a lot of misinformation, and people in the middle of that misinformation and the remoteness of the doctors (...) you don't know who to call, everyone begins to have an opinion, everyone lost, anguished	P4: Hay mucha desinformación, y las personas en medio de esa desinformación y la lejanía de los médicos (…) no sabes a quien llamar, todo el mundo empieza a opinar, todo el mundo perdido, angustiado
**Symptoms and secondary effects of cancer, identifying those at higher risk and addressing**	P1: The chemotherapy left very strong havoc on me ... And I'm trying to fix it by myself	P1: La quimioterapia dejó estragos muy fuertes en mi… Y los estoy tratando de arreglar yo
	P3: There have been hard times when the pain for the first time bent me over and made me physically cry for pain.	P3: Han pasado momentos duros en que el dolor por primera vez me dobló y me puso a llorar físicamente por dolor.
	P4: Occasionally I have insomnia, changes too ... emotional	P4: Ocasionalmente tengo insomnio, cambios también…Emocionales

**Priorities**	**Focus group participant quotes**	
	P2: As a result of chemotherapy and radiotherapy, your balance affects you, so there are times when I feel like I'm going to fall. If I am on my feet for a while, I feel that I am going sideways it has affected me, my hearing, my memory, now I forget things a lot, and the consequences are difficult	P2: A raíz de la quimioterapia y radioterapia, le afecta a uno el equilibrio, entonces yo hay veces que siento que me voy a caer. Si yo estoy de pie un tiempo, siento que me voy de medio lado, me ha afectado, el oído, la memoria, ahora se me olvidan mucho las cosas, y las secuelas son duras
	P3: I was warned about the side effects of chemotherapy, in a way, quite abrupt, which at that time was super shocking (...) It is not so much that they do not inform you, but how they inform you	P3: A mí me advirtieron los efectos secundarios de la quimioterapia, de una forma, bastante brusca, que en ese momento fue super impactante (…) No es tanto que no te lo informen, sino como te lo informan
**Identifying regional differences and socioeconomic inequalities in cancer survival to improve cancer care and outcomes in vulnerable populations**	P2: I talk to many people and tell them something about my story. I recommend (...) do not hesitate to ask your doctor to order a specialized exam (...) If I (...) had ordered a colonoscopy, I know which, for an EPS, does not cost millions. How much would I have avoided myself in life?	P2: Yo hablo con mucha gente y les cuento algo de mi historia, les recomiendo (…) no dude en pedirle a su médico que le ordene un examen especializado (…) Si a mí (...) me hubiera ordenado una colonoscopia, que sé que, para una EPS, no le cuesta millones. ¿Cuánto me hubiera evitado yo en la vida?
	P2: The EPS, are as very left in the forgetfulness of the patient	P2: Las EPS, están como muy dejadas en el olvido del paciente
	P3: Why is there a classification in the patient's range if we are all human beings? So, I treat you this way, and you another, that should not be so! And that should not happen in our health system.	P3: ¿Por qué hay una clasificación en el rango del paciente si todos somos seres humanos? Entonces a ti te trato de esta manera, y a ti de otra, ¡eso no debería ser así! Y eso no debería pasar en nuestro sistema de salud.
	P2: To cure me (...) every third day I had to travel from my home to the clinic (...) I would have been spending between an hour and a half and two hours traveling. So, just imagining the route I had to take, I was surely going to die.	P2: Para hacerme curaciones (...) cada tercer día desplazarme desde mi vivienda hasta la clínica (...) hubiera estado, gastando entre hora y media y dos horas de recorrido. Entonces, solamente con imaginarme el recorrido que tenía que hacer, seguramente me iba a morir.
	P2: Medicines are a terrible fight (…) claiming medicines and, unfortunately, for them, there is always an inconsistency! (…) You can wait, 15 minutes, 20 minutes. But why one hour, why two hours?	P2: Los medicamentos es una lucha terrible (…) reclamar los medicamentos y siempre desafortunadamente, ¡para ellos hay una inconsistencia! (…) puede esperar, 15 minuticos, 20 minuticos. ¿Pero por qué una hora, porque dos horas?
	P1: Going to settle the disability (…) waiting to be paid, then they tell me that they deny them. So, I am right now at the guardianship level	P1: Ir a radicar la incapacidad (…) esperando a que me las pagaran, luego me dicen que, las niegan. Entonces yo estoy ahorita a nivel de tutela
	P3: They denied me all disabilities. They have paid me absolutely nothing. And the payments are always, so late.	P3: Me negaron todas las incapacidades, no me han pagado absolutamente nada. Y los pagos siempre son, super atrasados.
**Health promotion and encouraging lifestyle change**	P1: Eat everything and, you will see that you will live many years, and I have followed your advice, and definitely, good nutrition has stood up	P1: Coma de todo y verá que va a vivir muchos años, y he seguido su consejo, y definitivamente, la buena alimentación me tiene de pie

Priorities	Focus group participant quotes	
	P4: A theme like food is a theme that does not tell you anything, and information is reaching you everywhere	P4: Un tema como la alimentación es un tema que tampoco te dicen nada y te está llegando información por todo lado
	P2:EPS had a stress management program	P2: Las EPS, tuvieran un programa de manejo del estrés
**Providing psychosocial support after cancer treatment**	P1: You refuse to live because you have the cancer ghost	P1: Uno mismo se niega de vivir, porque tiene el fantasma del cáncer
	P2: Unfortunately, cancer is synonymous with death. I was not prepared for that	P2: Desafortunadamente, cáncer es sinónimo de muerte, uno no está preparado para eso
	P3: Definitely, emotional support, not only for the patient but for his family system	P3: Definitivamente, el apoyo emocional, no solamente para el paciente, sino para su sistema familiar
	P1: He most that my doctor is my friend, every time I have a consultation with him, I tell him I love him, I love him, because I stand up, thanks to you. I leave that office and I forget that I have chronic cancer, that I may never be cured, because he gives me strength, he gives me faith, he gives me hope.	P1: El más que mi médico es mi amigo, yo cada vez que tengo consulta con él, le digo lo amo, lo quiero, porque yo estoy de pie, gracias a usted. Yo salgo de ese consultorio y se me olvida que tengo cáncer crónico, que puede que nunca me vaya a curar, porque el me da la fuerza, me da la fe, me da la esperanza.
	P1: He put me in psychiatric and psychological therapy treatment because he told me, I need you to heal your soul, you heal your heart and your interior. So that we can beat cancer.	P1: Me puso en un tratamiento de terapia psiquiátrica y psicológica, porque me dijo, necesito que para que se sane tu alma, sanes tu corazón y tu interior. Para que podamos vencer el cáncer.
	P4: There really is a disparity with the people of your family member, or simply, you do not feel comfortable that your family is poor you ... because you do not want them to see you as a sick person	P4: Realmente hay disparidad con las personas de tu familiar, o simplemente, no te sientes cómodo con que tu familia te esté pobreteando...Porque no quieres que te vean como un enfermo
**Care co-ordination to increase patient access and integration of cancer care**	P3: I have always been more of … Towards the inclination of medicines, which are known as alternatives ... But they are not really alternatives but are complementary and integrative.	P3: Siempre he sido más de… Hacia la inclinación de las medicinas, que se conocen como alternativas... Pero que realmente no son alternativas, sino son complementarias e integrativas.
	P4: Because they do not join a nutritionist, they do not join a person who helps you reduce stress, because stress generates more cancer cells, etc. An interdisciplinary team that handles all these issues. Well, because the doctor is an oncologist and is focused on all these issues, he knows and is specialized. But, there must be an interdisciplinary team to guide you, which is many of the issues that happen and what happens ... People start looking on their own	P4: Porque no unen un nutricionista, no unen una persona que te ayude a bajar el estrés, porque el estrés, te genera más células cancerosas, etc. Un equipo interdisciplinario que maneje todos estos temas. Pues porque el médico si es oncólogo y está centrado en todo estos temas, sabe y está especializado. Pero, debe haber un equipo interdisciplinario que te guie, que es muchos de los temas que suceden y que pasa… La gente empieza a buscar por su cuenta
	P4: We have talked about the importance of having an internist in the process. Because an internist sees himself as a whole and that internist could be the coordinator of your process	P4: Hemos hablado de la importancia que haya un internista en el proceso. Porque un internista se ve como un todo, y ese internista, podría ser el coordinador de tu proceso

Priorities	Focus group participant quotes	
	P1: I was seen by the oncologist, on the other hand, the dermatologist, on the other hand, the rheumatologist on the other hand (…) that they do not see us as sick patients, but as human beings who need to be rescued!	P1: A mí el oncólogo, me veía por aparte, el dermatólogo por otro lado, el reumatólogo por otro lado (…) que no nos vean como pacientes enfermos, ¡sino como seres humanos que necesitamos ser rescatados!

Abbreviations: EPS: Entidades Promotoras de Salud; P1: Woman diagnosed with Non-Hodgkin Lymphoma in 2014, remission in 2017, and relapsed in 2019 in treatment with chemotherapy. P2: Man diagnosed with Colon cancer in 2017 with chemotherapy and multiple surgeries. P3: Woman diagnosed with Breast cancer in 2017, relapsed in 2019 with immunotherapy, chemotherapy, and alternative medicine, P4: Woman diagnosed with Breast cancer with chemotherapy, radiotherapy, and surgery.

Table S3. Prioritization survey questions.

**Prioritization survey**	
**This questionnaire seeks to determine the prioritization of the most relevant needs in patients living with and over cancer in Colombia. The questionnaire is made up of 2 parts, answer with the greatest sincerity of the case and remember that there are no wrong questions.**	
**Part 1**	
1. What is your profession or role in "living with and beyond cancer? Please select the one that you consider to be most relevant to the exercise of identifying priorities in" living with and beyond cancer".<br>- Patient/Cancer survivor<br>- General physician/clinician<br>- Specialist at any area related to cancer treatment (oncologist, surgeon, radiotherapist, etc.)<br>- Specialist at palliative care<br>- Nursing<br>- Psychiatrist or phycologist<br>- Other allied health professionals (physiotherapist, nutritionist, etc.)<br>- Researcher<br>- Engineer<br>- Artist, Master of Arts<br>- Administrative (relate to the care of cancer patients)<br>- Other, please describe	
**Professionals**	**Patients and survivors**
2. How many years of experience do you have in oncology?<br>- Less than two years<br>- Two to five years<br>- Five to ten years<br>- More than 10 years<br>- None of the above	2. In what year were you diagnosed with cancer?<br>3. At this time, what type of treatment or care are you receiving? you can select multiple answers.<br>- I am not receiving treatment or care related to my cancer<br>- Chemotherapy<br>- Hormonotherapy<br>- Radiotherapy<br>- Immunotherapy<br>- Palliative care<br>- Alternative treatment<br>- Other, please describe
**Part 2**	
1. Please rank the following priorities according to their importance to patients who are living with and beyond cancer. Indicate with 1 the most important, with 6 the least important. If you consider that the statement is not relevant select N / A<br><br>- Provide a list of information re-sources and available support services for managing long-term cancer care to patients and caregivers.<br>- Explain expected symptoms and secondary effects of cancer to the patients, identifying those for which the patient is at higher risk.	

**Prioritization survey**
- Identifying regional and socioeconomic differences inequalities in cancer survival and formulate plans to improve cancer care with a differential focus for vulnerable populations.<br>- Health promotion and encouraging lifestyle change for patients and survivors.<br>- Providing psychosocial support before, during, and after cancer treatment.<br>- Care coordination to increase patient access and integration of cancer care.
2. If you want to add another priority for this exercise, please comment it here, and indicate its prioritization (very high, high, intermediate, or low)
Thank you for your participation! On behalf of the organizing committee of "Living with and beyond cancer".

Table S4. Prioritization survey questions - Spanish.

**Cuestionario de priorización**	
**El presente cuestionario busca determinar la priorización de las necesidades más relevantes en pacientes que viven con y más del cáncer en Colombia. El cuestionario está conformado por 2 partes, responda con la mayor sinceridad del caso y recuerde que no hay preguntas equivocadas.**	
**Parte 1**	
**1.** ¿Cuál es su profesión o rol en "vivir con y más allá del cáncer"? Por favor, seleccione el que usted considera es el más relevante para el ejercicio de identificar prioridades en "vivir con y más allá del cáncer".<br>- Paciente/sobreviviente de cáncer<br>- Médico general<br>- Médico especialista en algún área relacionada con el tratamiento del cáncer (oncólogo, cirujano, radioterapeuta, etc.)<br>- Médico especialista en cuidados paliativos<br>- Enfermero<br>- Psiquiatra - psicólogo<br>- Otros profesionales de la salud (fisioterapia, nutricionista, etc.)<br>- Investigador<br>- Ingeniero<br>- Artista/ maestro en bellas artes<br>- Administrativo (relacionado con cuidado para pacientes con cáncer)<br>- Otro, por favor especifique	
**Profesionales**	**Pacientes y sobrevivientes**
2. ¿Cuántos años de experiencia tiene en el área de oncología?<br>- Menos de dos años<br>- Dos a cinco años<br>- Cinco a 10 años<br>- Más de 10 años<br>- Ninguno de los anteriores	2. ¿En qué le diagnosticaron con cáncer?<br>3. ¿En este momento, qué tipo de tratamiento o cuidado recibe? Puede seleccionar múltiples respuestas.<br>- No estoy recibiendo tratamiento/cuidado relacionado con mi cáncer en este momento<br>- Quimioterapia<br>- Hormonoterapia<br>- Radioterapia<br>- Inmunoterapia<br>- Cuidados Paliativos<br>- Tratamientos alternativos<br>- Otro, por favor indique
**Parte 2**	
1. Por favor, ordene las siguientes prioridades según su importancia para pacientes quienes están viviendo con y más allá del cáncer. Indique con 1 el más importante, con 6 el menos importante. Si usted considera que el enunciado no es relevante seleccione N/A<br><br>- Proveer al paciente o cuidador un listado de recursos de información y servicios de apoyo disponibles para administrar la atención del cáncer a largo plazo<br>- Explicar al paciente los síntomas y efectos secundarios del cáncer, especificando aquellos con mayor riesgo para el paciente. Adicionalmente proveer información sobre el manejo de síntomas<br>- Identificar las desigualdades regionales y socioeconómicas en la atención y supervivencia del cáncer y formular planes de mejora, con un enfoque diferencial para poblaciones vulnerables.	

Cuestionario de priorización
- Promover la salud y fomentar cambios en los estilos de vida de pacientes y supervivientes.<br>- Brindar apoyo psicosocial antes, durante y después del tratamiento del cáncer.<br>- Coordinar la atención para aumentar el acceso de los pacientes y la integración de la atención del cáncer.
2. Si usted quiere añadir otra prioridad para este ejercicio, por favor coméntelo aquí, e indique su priorización (muy alta, alta, intermedia, o baja).
¡Muchas gracias por su participación! En nombre del comité organizador de "Vivir con y más allá del cáncer".
